# Influenza H1N1pdm-specific maternal antibodies offer limited protection against wild-type virus replication and influence influenza vaccination in ferrets

**DOI:** 10.1111/irv.12220

**Published:** 2013-12-16

**Authors:** Amorsolo L Suguitan, James R Zengel, Scott Jacobson, Stephanie Gee, Janet Cetz, Paulyn Cha, Zhongying Chen, Rosemary Broome, Hong Jin

**Affiliations:** MedImmuneMountain View, CA, USA

**Keywords:** Ferrets, immunogenicity, influenza, live attenuated influenza vaccine, maternal antibodies, passive immunity

## Abstract

**Objective:**

The objective was to study passively acquired influenza H1N1 pandemic (H1N1pdm) maternal antibody kinetics and its impact on subsequent influenza infection and vaccination in ferrets during an outbreak of the H1N1pdm.

**Design and main outcome measures:**

Infectivity of the H1N1pdm in the respiratory tract of ferrets was compared with the previous seasonal A/South Dakota/6/2007 (SD07, H1N1). Influenza-specific antibodies were quantitated and antibody-mediated protection against the homologous and heterologous H1N1 virus challenge infection was determined.

**Results:**

H1N1pdm virus was approximately 10 times more infectious than SD07 in ferrets, replicated to higher viral titers in the upper respiratory tract and shed for a longer duration. Influenza-specific antibodies after natural infection persisted much longer in the circulation than passively acquired maternal antibodies. The protection conferred by the maternal antibodies was limited to the homologous virus strain and was ineffective against SD07 and H3N2 virus. Serum antibodies from maternal transmission or passive transfer interfered with homologous vaccine strain-mediated antibody responses in the ferret. A booster immunization was required to elicit a high level of antibody.

**Conclusions:**

The findings support the rationale for a prime and boost immunization strategy in young children in whom maternal antibodies are present.

## Introduction

Influenza virus is a significant cause of respiratory tract infections resulting in an estimated 3–5 million clinical infections and up to half million deaths annually worldwide.^1^ Approximately 1 million children suffer from severe cases of influenza with 100 000 deaths annually.^2^ The 2009 swine-origin influenza A H1N1 pandemic (H1N1pdm) spread to more than 200 countries worldwide and caused half a million deaths during the first year of its circulation.^3^ The antigenicity of this swine-origin virus is distinct from previously circulating seasonal H1N1 viruses.^4^ It caused acute infection mainly in children and younger adults, while older adults were mostly resistant to H1N1pdm infection, presumably due to cross-reactive antibodies from previous exposure to antigenically related strains.^5^–^7^

Pregnant women^8^ and infants <6 months old are more likely to experience severe complications from influenza infection,^9^,^10^ and pregnancy was a significant risk factor for H1N1pdm-related morbidity and mortality.^11^,^12^ There are currently no licensed influenza vaccines or antiviral drugs approved for infants <6 months old.^2^,^13^ Indirect protection via maternal vaccination and vaccination of all individuals with close contact with neonates are recommended measures to prevent infection in early infancy.^14^ However, the level and half-life of maternal antibodies varies, and it is difficult to define the duration of protection conferred by maternal antibodies against different infectious agents.^15^ The high levels of pre-existing antigen-specific maternal antibodies could significantly inhibit the immunogenicity of some vaccines.^16^–^18^ Thus, immunizations are often scheduled when passively acquired antibodies have waned.

Ferrets are naturally susceptible to infection with a wide variety of influenza viruses and display symptoms similar to that seen in humans, which make them an attractive model to study influenza.^19^,^20^ However, this natural susceptibility to such a ubiquitous virus makes keeping a ferret colony influenza-free a challenge. In 2011, the H1N1pdm virus caused an influenza outbreak in a ferret breeding colony. The outbreak was attributed to a colony caretaker who had returned to work too soon after contracting influenza virus. The outbreak was explosive: Within a day of noting the first ferret sneezing, clinical signs were observed throughout the colony of over 300 animals of varying ages, including pregnant dams and newborn kits. In the present study, we evaluated the infectivity and morbidity caused by the H1N1pdm virus in comparison with a previously circulating seasonal H1N1 strain, A/South Dakota/6/2007 (SD07), to understand why the H1N1pdm virus caused such rapid spread in the colony. In addition, we used surgically implanted transponders that continuously monitored ferret body temperature to obtain an accurate depiction of the fever induced by influenza infection.^21^,^22^ The influence of passively transferred maternal antibodies on protection against homologous and heterologous viruses and their influence on the immunogenicity of live attenuated influenza vaccines (LAIV) in young kits were also investigated.

## Materials and methods

### Viruses

Wild-type influenza A/South Dakota/6/2007 (SD07, H1N1), A/Rhode Island/1/2010 (RI10, H3N2), and A/California/7/2009 (CA09, H1N1pdm) were kindly provided by the Centers for Disease Control and Prevention (CDC, Atlanta, GA, USA). Influenza A/Gilroy/231/2011 (Gil11, H1N1pdm) was isolated from the nasal wash of a symptomatic ferret. The HA and NA genes of Gil11 are closely related to CA09 virus, and their sequences have been deposited in GenBank (accession # KC436084 for HA and # KC436085 for NA). The influenza viruses were expanded in either Madin–Darby canine kidney (MDCK) cells or 10- to 11-day embryonated specific pathogen-free hen eggs at 37°C.

### H1N1 virus infectivity in ferrets

All animal experiments were conducted in accordance with Institutional Animal Care and Use Committee (IACUC)-approved protocols. Groups of 3 ferrets, between 8 and 10 weeks old and confirmed seronegative for influenza-virus-specific antibodies, were intranasally inoculated with 10-fold dilutions ranging from 1 to 10^5^ plaque-forming unit (PFU) of Gil11 or SD07 virus cultured in MDCK cells.^23^ Gil11 grown in eggs had L191I change in the HA. Ferrets were considered infected if the virus was detected in the nasal wash or if influenza-specific antibodies were detected at the end of the study on day 9 post-infection (p.i.). The FID_50_ was calculated using the method described by Reed and Muench.^24^

### Morbidity induced by H1N1pdm virus infection in ferrets

One week prior to infection, groups of 6-week-old ferrets (*N* = 4) from Simonsen Laboratories (Gilroy, CA, USA) were surgically implanted with a small transponder (DSI, St. Paul, MN, USA) intraperitoneally that transmits core body temperature information via telemetry over 5-minute intervals. As Gil11 did not replicate well in MDCK cells and eggs, the CA09, which is antigenically similar to Gil11, was used instead in this study. The ferrets were then infected intranasally with PBS (mock-infected), 10^7^ TCID_50_ of CA09 or 10^7^ TCID_50_ of SD07 virus. Weight measurements and nasal wash collections were performed on days 1, 2, 3, 5, 7, and 9 p.i. Each infected ferret was monitored and scored for influenza-like illness or other clinical symptoms such as sneezing, lethargy, and the presence of nasal or ocular discharge. Virus titers in the nasal washes were measured by TCID_50_ in MDCK cells.

### Serum HAI antibody kinetics in ferrets

Serum samples were collected bi-weekly from 24 H1N1pdm-infected adults (average age of 33 weeks), 18 infected kits (average age of 4 weeks) for up to 26 weeks after the outbreak, and 22 kits that were born 4 weeks after the outbreak and were weaned from previously infected dams with collection beginning after weaning (average of 4 weeks). Hemagglutination inhibition (HAI) assay was used to determine H1N1pdm-specific serum antibody levels as previously described.^25^

### Influence of serum antibodies on subsequent viral infection and vaccination

Groups of 4 age-matched (approximately 6 weeks) naïve ferrets and kits with passively acquired maternal H1N1pdm HAI antibodies (titer of 32–128) were infected with 10^5^ plaque-forming units (PFU) of Gil11 (due to its low titer), 10^7^ PFU of SD07, or 10^7^ PFU of RI10. The ferrets were sacrificed on day 3 p.i., and their lungs and nasal turbinates were harvested, homogenized, and titrated on MDCK cells by TCID_50_. Virus titers were calculated using the Reed and Muench method.^24^ Groups of 4 age-matched (approximately 6 weeks) naïve ferrets and kits with maternal anti-H1N1pdm HAI antibodies (titer of 64–128) were immunized intranasally with the 2011–2012 seasonal LAIV consisting of 10^7^ fluorescent focus units (FFU) each of A/California/7/2009 (H1N1pdm), A/Perth/16/2009 (H3N2) and B/Brisbane/60/2008 on days 0 and 28. Serum samples were collected on days 28 (post-dose 1) and 56 (post-dose 2), and HAI antibody titers were determined against the corresponding wild-type viruses of each of the three vaccine strains as described earlier.

The impact of serum antibody on vaccination was further evaluated with passively transferred ferret hyperimmune serum. Normal ferret serum, undiluted hyperimmune ferret serum against CA09 with HAI titer of 2048, or a 1:8 dilution of hyperimmune ferret serum in a volume of 1·0 ml was administered intravenously (i.v.) to groups of 6-week-old ferrets (*n* = 4). After confirming the seropositive status of the passively immunized ferrets by HAI (titer of 32–64 for those with undiluted hyperimmune serum and 4–8 among those that received the diluted serum), the kits were immunized with the 2011–2012 seasonal LAIV and serum samples were collected post-dose 1 and post-dose 2, and HAI antibody titers were determined as previously described.

## Results

### H1N1pdm is more infectious and pathogenic than seasonal H1N1 in ferrets

To determine whether the rapid spread of an influenza H1N1pdm-like virus among ferrets could be explained by its high infectivity in these animals, Gil11 H1N1pdm was compared with the seasonal SD07 H1N1 virus for infectious dose at which fifty percent of ferrets could be infected (FID_50_). As shown in Table 1, the Gil11 virus was highly infectious in ferrets, with an FID_50_ of 3·2 PFU which was 10-fold lower than SD07 H1N1 (32 PFU). The small sample size precludes statistical analysis of the FID50 studies, but these results are comparable to the FID_50_ values of other H1N1pdm that we have examined (data not shown).

**Table 1:** Infectivity of H1N1 viruses in ferrets

Virus	Virus dose (PFU)	No. seroconverted/total*	No. shedding/total	FID_50_ (PFU)	Viral titer in nasal wash**
A/Gilroy/231/2011 (H1N1pdm)	1	0/3	0/3	3·2	<1·2
	10	3/3	2/3		3·3
	100	3/3	3/3		3·3
A/South Dakota/6/2007 (H1N1)	10	1/3	1/3	32	2·2
	100	2/3	2/3		3·6
	1000	3/3	3/3		5·7

* Seroconversion was determined by HAI assay.

** Mean peak viral titer expressed as TCID_50_/ml.

**Figure 1:**
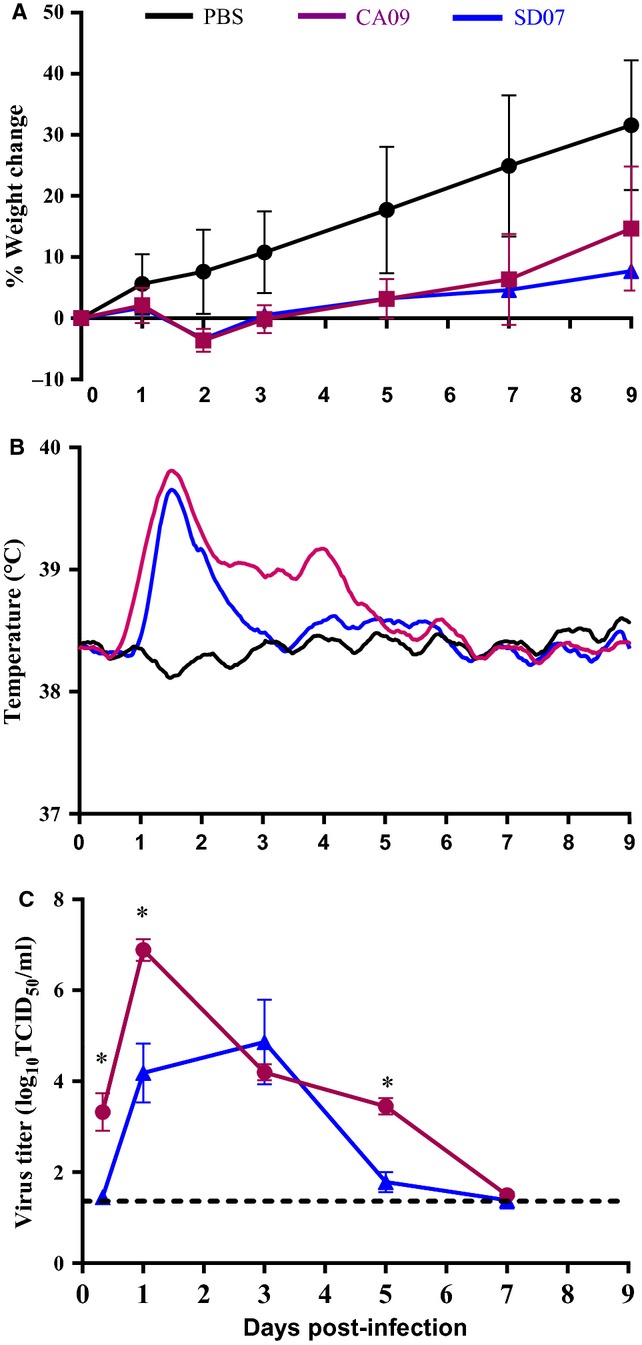
Morbidity induced by H1N1 viruses in ferrets. Groups of four 8-week-old male and female ferrets were inoculated intranasally with PBS, 10^7^ TCID_50_ of A/California/7/2009 (CA09; H1N1) or 10^7^ TCID_50_ of A/South Dakota/6/2007 (SD07; H1N1) wild-type virus. Mean percentage change in body weight post-infection is depicted in (A), the Lowess curves of the mean core body temperatures measured via telemetry are summarized in (B), and mean virus titer in the nasal wash reported as TCID_50_/ml is shown in (C). The dashed line in (C) indicates the assay's limit of detection. An asterisk designates statistical significance between the mock- and virus-infected groups that are being compared (*P* < 0·05, Mann–Whitney *U*-test).

H1N1pdm09 viruses generally replicated more efficiently in the lungs than previous seasonal H1N1 viruses and caused greater morbidity in infected ferrets.^26^–^28^ Similar to other swine-origin influenza H1N1pdm 2009 viruses, Gil11 replicated poorly in both MDCK cells and eggs, reaching a peak titer of 10^5^ TCID_50_/ml only.^29^ Gil11 was antigenically similar to CA09 as determined by serum HAI assay.^30^ CA09 can grow to high titer after egg adaptation and was therefore used to evaluate the morbidity caused by H1N1pdm infection in ferrets instead of Gil11.^25^ The ferrets that were infected with either CA09 or SD07 virus showed typical symptoms of influenza infection including weight loss, elevated body temperature, and lethargy. All ferrets (4/4) infected with CA09 started sneezing on day 2 p.i. and continued until day 6 p.i., while only half (2/4) of ferrets infected with SD07 displayed sneezing, with an onset on day 6 p.i. CA09- and SD07-infected ferrets displayed similar patterns of weight loss, losing about 4% of body weight on day 2 p.i. and quickly rebounding by day 3 p.i. (Figure 1A). However, compared with the mock-infected group, there was a pronounced delay in the weight gain experienced by ferrets infected with either H1N1 virus. Both CA09- and SD07-infected ferrets experienced elevated body temperatures that peaked between days 1 and 2 p.i. (Figure 1B) as measured accurately by the implanted thermometers. Interestingly, each CA09-infected ferret exhibited a biphasic temperature curve with a longer duration of fever with a secondary peak between days 4 and 5 p.i. In contrast, the body temperature of SD07-infected ferrets declined to the baseline level by day 3 p.i. CA09 replicated rapidly in the upper respiratory tract of ferrets at a peak titer of 6·8 log_10_ TCID_50_/ml on day 1 p.i and declined until day 7 p.i. (Figure 1C). The peak titer of SD07 was 4·8 log_10_ TCID_50_/ml on day 3 p.i. (100-fold lower than CA09) and was mostly cleared by day 5 p.i. Taken together, these results suggested that although SD07 and CA09 caused similar weight loss in young ferrets, CA09 induced a longer duration of fever, replicated rapidly to a higher level, and then showed delayed clearance in the upper respiratory tract as compared to SD07.

### Kinetics of influenza-specific antibodies from natural infection and passive immunity in ferrets

**Figure 2:**
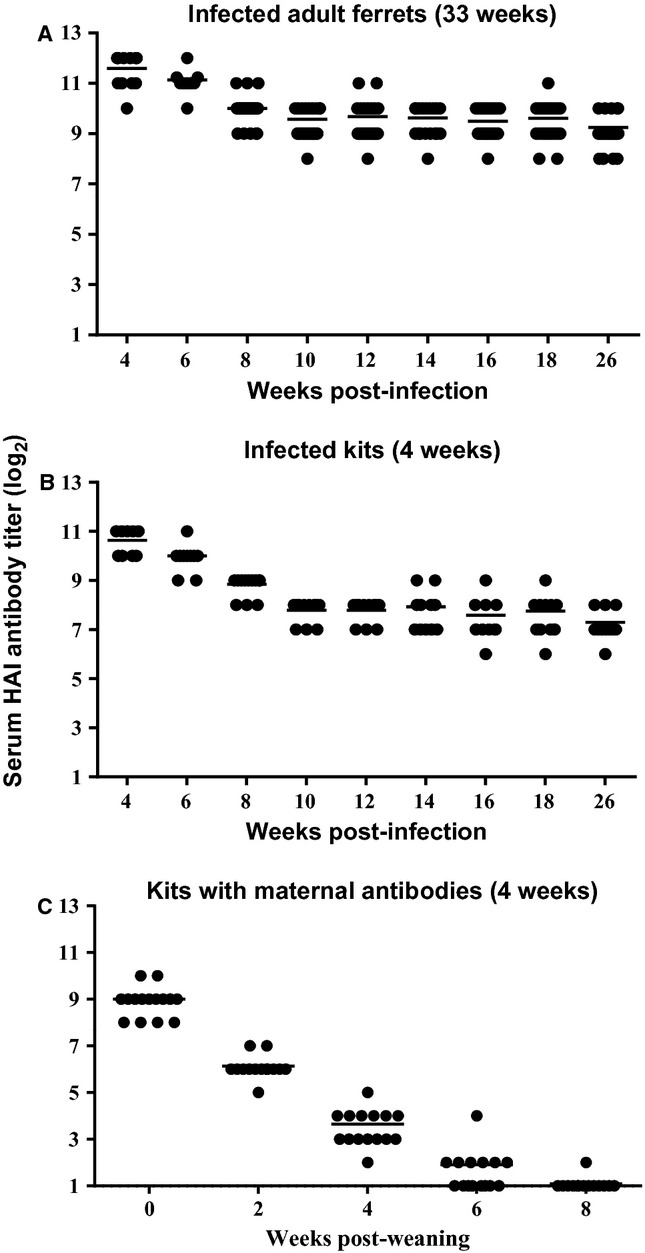
Kinetics of antibody decay among naturally infected ferrets and kits with passively derived maternal anti-H1N1 HAI antibodies. Serum samples from (A) adult (average age of 33 weeks on onset of outbreak) (*n* = 24) and (B) kits (average age of 4 weeks) (*n* = 18) that were naturally infected with influenza A H1N1 virus, as well as from (C) kits (average age of 4 weeks) (*n* = 22) that were weaned from dams that were previously infected with H1N1, were collected bi-weekly 4 weeks after the H1N1 outbreak in the ferret breeding colony and anti-CA09 (H1N1) serum HAI antibody titers were determined.

Two groups of ferrets, adult ferrets (average age of 33 weeks) and young kits (average age of 4 weeks), were identified as naturally infected with Gil11 during the outbreak by clinical symptoms such as sneezing and lethargy, which were subsequently confirmed by serum HAI antibody titers against CA09 (titer of 1024–4096) (Figure 2A,B). Serum HAI antibodies of naturally infected adults and kits had similar kinetics; the antibodies declined by approximately four- to eight-fold from 4 to 10 weeks p.i. and remained at that level for both adults (256–1024 or 8–10 log_2_) and kits (64–512 or 6–9 log_2_). In comparison, kits born after the outbreak from Gil11-infected jills also had high HAI titers of 256–1024 (8–10 log_2_) at 4 weeks of age, but exhibited a more rapid decline in maternal antibody. These antibody levels were barely detectable 6 weeks post-weaning (Figure 2C). Thus, the HAI antibodies from natural infection decayed slower in the circulation than the maternal antibodies.

**Figure 3:**
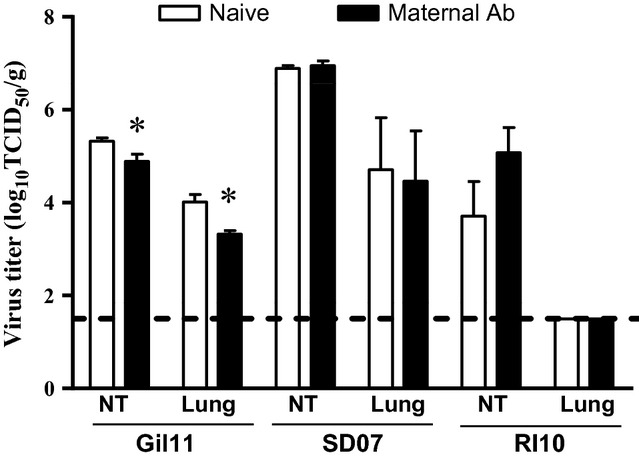
Protection conferred by passive maternal H1N1pdm HAI antibodies. Groups of four 6-week-old naïve ferrets and kits with passively acquired maternal HAI antibodies (titer of 16–64) were infected with either 10^5^ TCID_50_ of the homologous A/Gilroy/231/2011 (H1N1pdm), 10^7^ PFU of A/South Dakota/6/2007 (SD07; H1N1), or 10^7^ PFU A/Rhode Island/1/2010 (H3N2) virus. The ferrets were sacrificed on day 3 p.i., and virus titers in the nasal turbinates and lungs were determined and expressed as TCID_50_/g of tissue. Dashed lines indicate the assay's limit of detection, while an asterisk designates statistical significance between the groups of naïve ferrets and those with maternal antibodies that are being compared (*P* < 0·05, Mann–Whitney *U*-test).

The role of these maternal antibodies in protection against infection with H1N1 and H3N2 viruses was investigated. Groups of age-matched (approximately 6 weeks) naïve ferret kits and kits with maternal HAI antibodies (titer of 64–128) were infected with the homologous Gil11 virus grown in MDCK cells, SD07, or RI10 grown in eggs, respectively. When infected with homologous Gil11 virus, there was a slight but significant reduction in viral burden in the lung (2·8-fold) and NT (4·9-fold) of kits with maternal antibodies compared with the control group (Figure 3). In contrast, replication of the SD07 (H1N1) and RI10 (H3N2) viruses was not different between the naïve and H1N1pdm-seropositive ferrets. Thus, the protection conferred by influenza-specific maternal antibodies was limited and highly strain-specific.

### Influence of maternal antibodies on influenza vaccine immunogenicity

**Figure 4:**
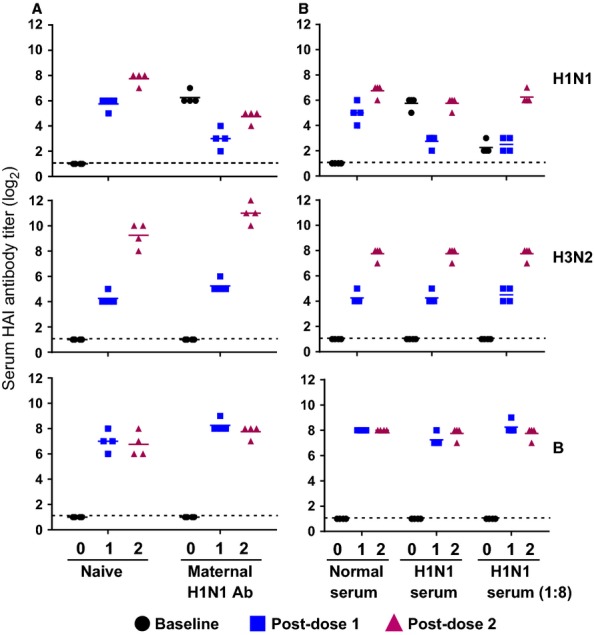
Influence of passive maternal anti-H1N1 HAI antibodies on vaccine immunogenicity. Groups of 4 age-matched naïve ferrets and those with maternal H1N1 HAI antibodies (titer of 64–128) (A), passively transferred normal or H1H1pdm ferret serum either undiluted or 1:8 diluted (B), were immunized with the 2010–2011 LAIV intranasally on day 0 and day 28. Serum HAI antibody titers to each of the homologous wild-type viruses in the vaccine were measured on day 0 (baseline), day 28 (Post-dose 1), and day 56 (Post-dose 2).

Groups of age-matched naïve kits and those with maternal antibodies (HAI titer of 64–128) were immunized with the 2011–2012 seasonal live attenuated influenza vaccine (LAIV) on day 0 and boosted 4 weeks later. A single dose of LAIV in naïve ferrets was immunogenic and elicited high HAI antibody responses to each of the three vaccine components and was boosted by a second dose (Figure 4A). LAIV elicited a similar pattern of HAI antibody responses against the H3N2 and B strains in the kits with maternal antibodies but required two doses to induce H1N1pdm-specific antibody responses, and the level was not as high as that observed in naïve ferrets. These results indicate that the maternal antibodies that cross-react with the immunizing influenza strain could dampen the humoral immune response against that strain.

To confirm the reproducibility of these results, normal ferret serum, undiluted hyperimmune ferret serum against CA09 (HAI titer of 2048, or 11 log_2_), and a 1:8 dilution of hyperimmune ferret serum were passively administered i.v. to age-matched naïve kits to mimic the serostatus of kits with existing maternal antibodies. The serum HAI titers in these ferrets were confirmed to be comparable to those with maternal antibodies (titer 32–64 for undiluted hyperimmune serum recipients; 4–8 for the diluted serum group). The ferrets that received the hyperimmune serum had very low H1N1-specific HAI antibody responses post-dose 1, which was boosted by the 2nd dose (Figure 4B). The HAI antibody titers to H3N2 were initially low (titer of 16, or 4 log_2_) but were significantly boosted after the 2nd dose. B vaccine strain was robust after dose 1 (HAI titer of 256, or 8 log_2_) and was not boosted by a 2nd dose. This study further supports the finding that influenza-specific maternal antibodies influenced the homologous vaccine-strain-mediated antibody response.

## Discussion

Influenza H1N1pdm 2009 viruses are more infectious than previously circulating human seasonal H1N1 viruses in ferrets. The enhanced infectivity has been attributed to the neuraminidase and matrix proteins that are involved in particle release from the infected cells and viral morphology.^23^ Both H1N1pdm and seasonal H1N1 viruses are transmitted with similar efficiency via aerosol or respiratory droplets.^28^ The higher infectivity, higher virus yield, and/or greater expulsion of virus-containing particles of H1N1pdm in ferrets may explain the rapid spread of the infection in humans during the 2009 pandemic and the explosive outbreak of influenza in a ferret colony in 2011. It is speculated that the markedly widespread clinical symptom of sneezing in the ferret colony outbreak and the longer duration of virus shedding from H1N1pdm infection may have contributed to its efficient transmission.

Neither Gil11 nor CA09 was lethal in the ferrets that were naturally or experimentally infected with either virus, and none of the Gil11-infected pregnant dams were observed to have miscarriages during the outbreak. Although CA09 and SD07 induced similar clinical symptoms in ferrets, CA09-infected ferrets shed more virus in nasal washes than SD07-infected ferrets. The body temperature data generated by telemetry clearly demonstrated that CA09-infected ferrets had a characteristic secondary fever peak that occurred between days 4 and 5 p.i. This biphasic temperature curve induced by an H1N1pdm virus has also been previously reported by others.^22^,^31^ While the first febrile peak correlated with peak virus titers, the secondary febrile peak could be due to the innate response because CA09 is a more potent inducer of chemokines and cytokines than a seasonal H1N1 virus.^32^ The ferrets used in this study were in what is normally a rapid growth phase but the weight gain of experimentally infected animals was significantly lower than control animals as has been previously reported.^33^ The disease severity caused by H1N1pdm CA09 and Gil11 in the juvenile ferrets appeared to be less than what has been previously reported using older ferrets (5–9 months).^31^,^32^ The reasons are not clear, but viral sequence variations could be a factor.

The role of passively acquired maternal immunity to protection against influenza virus infection early in life was also investigated. Previous studies have indicated that ferrets are born with very low serum immunoglobulin levels and that majority of maternal antibodies are derived post-partum from the jill's colostrum and breast milk.^34^–^36^ The serum HAI antibodies in the kits born from the infected jills rapidly declined as soon as they were weaned and were barely detectable 6 weeks after weaning. In contrast, serum HAI antibodies of naturally infected adults and kits remained high 6 months after the outbreak, highlighting the difference in the persistence of antibodies gained through passive and active immunity. The protection mediated by maternal antibodies was highly subtype- and strain-specific.^34^ The protection was also limited as the reduction of challenge virus titers in the upper and lower respiratory tract was only 2·8- and 4·9-fold, respectively, compared with the naïve ferrets. It was possible that the protective effect was not very apparent due to the higher challenge virus dose used in this study. The ferrets with maternal antibodies lack heterosubtypic responses that are often mediated by the mucosal and cell-mediated immune responses evoked by natural infection.^37^,^38^ We did not examine mucosal antibody levels in naturally infected ferrets because the assay was not well established. It will be interesting to determine whether the protection was mediated by maternal IgG or IgA antibodies.

Although maternal influenza immunization is presumed to reduce influenza illness in infants <6 months old, maternal antibodies have been shown to negatively affect the immunogenicity of influenza vaccines in young chicks,^39^ foals,^40^ and pigs.^41^ This study is the first, to our knowledge, to investigate the influence of maternal antibodies on influenza vaccination in young ferrets. The maternal H1N1pdm antibodies reduced humoral responses to the H1N1pdm vaccine component in the LAIV, but not the responses to the influenza A H3N2 and B vaccine components. Maternal antibodies have also been associated with diminished humoral responses to other live attenuated vaccines against measles^42^ and rotavirus^43^ in humans. Understanding maternal antibody decay and its impact on vaccine immunogenicity may provide guidance in determining immunization schedules for very young infants with persisting maternal antibodies.
